# Breeding Sites of *Aedes aegypti*: Potential Dengue Vectors in Dire Dawa, East Ethiopia

**DOI:** 10.1155/2015/706276

**Published:** 2015-09-07

**Authors:** Dejene Getachew, Habte Tekie, Teshome Gebre-Michael, Meshesha Balkew, Akalu Mesfin

**Affiliations:** ^1^Department of Zoological Sciences, Addis Ababa University, P.O. Box 1176, Addis Ababa, Ethiopia; ^2^Department of Life Sciences, Dire Dawa University, P.O. Box 1362, Dire Dawa, Ethiopia; ^3^Aklilu Lemma Institute of Pathobiology, Addis Ababa University, P.O. Box 1176, Addis Ababa, Ethiopia

## Abstract

*Background and Objectives*. Entomological survey was carried out from May-June to September-October 2014 to investigate the presence of dengue vectors in discarded tires and artificial water containers in houses and peridomestic areas. *Methods*. A cross-sectional immature stage survey was done indoors and outdoors in 301 houses. Mosquito larval sampling was conducted using pipette or dipper depending on container types. Larvae were identified morphologically and larval indices were also calculated. *Results*. A total of 750 containers were inspected, and of these 405 were positive for mosquito larvae. A total of 1,873 larvae were collected and morphologically identified as *Aedes aegypti* (*n* = 1580: 84.4%) and *Culex* (*n* = 293: 15.6%). The larval indices, house index, container index, and breteau index, varied from 33.3 to 86.2, from 23.2 to 73.9, and from 56.5 to 188.9, respectively. *Conclusion*. *Aedes aegypti* is breeding in a wide range of artificial containers. To control these mosquitoes, the integration of different methods should be taken into consideration.

## 1. Introduction

Mosquito-borne diseases are the most significant public health risks globally [1]. Dengue fever infection is one of the most important arboviral diseases in humans [2]. It is endemic in Africa, the Americas, eastern Mediterranean, SE Asia, and the Western Pacific [2], threatening more than 2.5 billion people [2]. It is estimated that 50–100 million dengue infections occur each year [3]. Outbreaks exert a huge burden on populations, health systems, and economies in most tropical countries of the world [3]. Dengue viruses are the causative agents of dengue fever (DF) and dengue hemorrhagic fever/dengue shock syndrome (DHF/DSS) in humans [4, 5]. Arboviral infections are usually sensitive to changes in rainfall and temperature [4].

Population growth and increased individuals movement, urbanization, and the limited financial and human resources are attributed to the emergence and reemergence of the disease [6–8]. Presence of the virus, sufficient numbers of susceptible population, and mosquito vectors are required for dengue transmission [4, 6]. Reinfestation of vectors to new geographical areas, warm and humid climate, increased population density, water storage pattern in houses, storage of trash like tires, and introduction of new serotype of the virus serve as risk factors for dengue virus infections [9]. Travelers also have the potential to acquire and spread dengue virus infection [10].

*Aedes aegypti* and* Ae. albopictus* are the most important mosquito vectors of dengue fever viruses [11, 12].* Aedes aegypti* is the principal vector of dengue fever and dengue hemorrhagic fever in almost all countries [2, 13, 14].* Aedes africanus* and* Ae. luteocephalus* also act as potential vectors in Africa [14].* Aedes aegypti* and* Ae. albopictus* prefer laying their eggs in artificial containers [2] like flower vases, old automobile tires, buckets, and trash in general [6, 11, 15].

*Aedes aegypti* is the most efficient vector for arboviruses because it is highly anthropophilic, frequently bites, and thrives in close proximity to humans [13]. Infected* Ae. aegypti* [16] and* Ae. albopictus* [11] females may transmit the virus to their next generation transovarially.

The adult* Ae. aegypti* prefers to rest indoors and feed on humans during daylight hours [6]. Its peak biting periods are early in the morning and before dark in the evening [3, 6]. Once contracted the virus, the mosquito remains infected during its entire life and may transmit the virus during blood meals [16]. The viruses are maintained in an* Ae. aegypti*-human-*Ae. aegypti* cycle with periodic epidemics [6]. Most females of* Ae. aegypti* may spend their lifetime in or around the houses where they emerge as adults [13].

Though outbreaks of DF/DHF are poorly documented in Africa [17, 18], infections were also reported from eastern Africa [13, 18]. The prevention and control of dengue outbreaks mainly depend on the epidemiological surveillance of cases and mosquito vectors [19, 20]. Dengue is likely underrecognized and underreported in Africa because of low awareness by health care providers, other prevalent febrile illnesses, and lack of diagnostic testing and systematic surveillance [18]. Dengue morbidity can be reduced by implementing improved outbreak prediction and detection through coordinated epidemiological and entomological surveillance [3].

In Ethiopia, considerable but incompletely documented numbers of arboviral diseases are endemic [21]. However, infections remain underreported due to lack of laboratory facilities and inaccessibility of some of the endemic areas [21]. The disease was reported between 1985 and 1987 in refugees around Hargeysa in Somalia.

Dire Dawa is one of the two federal cities in Ethiopia and is called queen city of the desert. Different ethnic groups live in this city and there is high human mobility from neighboring countries like Djibouti and Somalia and from different parts of the world. Patients with fever, headache, abdominal discomfort, and diarrhea clinical symptoms were observed and confused with malaria and typhoid (Akalu Mesfin, Personal Comm.). At the end of 2013, in Dire Dawa, 9258 people were suspected of dengue fever (DF) and, of these, 40 were confirmed as dengue fever cases (Ethiopia Humanitarian Bulletin, unpublished data). The dengue fever that occurred in Dire Dawa varied from mild to severe with symptoms of sudden onset of fever which lasted for 2-3 days (extended to 4-5 days in some cases), headache (typically located behind the eyes), mild to severe muscle and joint pains (general body pain in some cases), feeling cold, and arthritis-like symptoms/pain. Nose bleeding and vomiting were also reported in few cases. Some of the patients were also hospitalized. According to the preliminary information gathered from some of the recovered patients, it was mentioned that they lose their appetites and feel weak especially after recovery.

Stored water in the container for long period, extended rainfall during the last rainy season, and ambient relative humidity and temperature may favor the breeding of* Ae. aegypti* and other aedine mosquitoes. Since the town is center of industry and tourism, it is preferred by people of neighboring countries (Djibouti and Somalia) for temporary stay during the hot season. Many people also migrate to the town in search of job linked with railway construction, and thus individuals infected with arboviruses such as dengue may disseminate the disease in the town aided by the bite of* Aedes* mosquitoes. Therefore in this study we tried to investigate and identify mosquito species' breeding in discarded tires and artificial water storage materials that serve as potential dengue fever transmission in Dire Dawa city. This study provides baseline information on the types of dengue virus vectors breeding in discarded tires and other artificial water containers in Dire Dawa, providing important information to the Federal Ministry of Health, city health bureau, and community of the city. Furthermore, the result of this study enables providing community awareness about the vectors and the protective measures to be taken. This study also provides the foundation for further investigation on dengue fever.

## 2. Materials and Methods

### 2.1. Study Area

The study was conducted in Dire Dawa city. Dire Dawa city is located at a distance of 515 km east of Addis Ababa, the capital city of Ethiopia. It is located at 9°35′35′′N and 41°51′57′′E with an altitude of 1191 masl. The municipality has a noncontinuous water supply (every 2 days) and irregular garbage collection. A house-to-house cross-sectional entomological survey was carried out in houses and peridomestic areas to detect mosquito larval breeding sites with a view to study the level of infestation of areas with* Aedes* larvae.

### 2.2. Sample Collection and Examination

Tires and artificial water containers were visually inspected for the presence of water and mosquito larvae and pupae from different randomly selected houses and tire repair storage sites. Each container was recorded for container type, location within the lot, sun exposure, lid status, water type, and water status.

**Figure 1:**
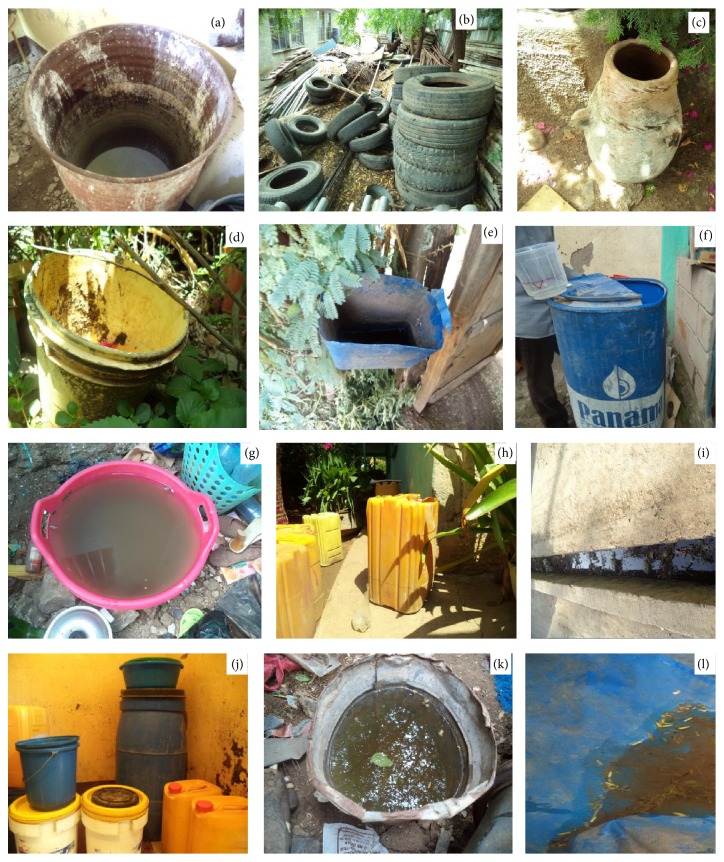
Mosquito larvae breeding in various habitats: (a) barrel, (b) tire, (c) mud pot, (d) plastic bucket, (e) dustbin, (f) plastic drum, (g) plastic bowel, (h) jerrican, (i) ditch, (j) containers in house, (k) bucket, and (l) polythene sheet.

The study was based on a cross-sectional entomological survey of tires and artificial water containers from May-June 2014 to September-October 2014 after rain. All containers (Figure 1) both indoors and outdoors which might harbor mosquito larvae and pupae were inspected to determine whether they were wet or dry and to check the presence or absence of mosquito larvae and pupae. Potential containers were counted and the 3rd stage and 4th stage mosquito larvae and pupae were collected.

Mosquito larvae were collected from discarded tires and other artificial containers with a plastic cup, pipette, or classical dipper. To decrease the effect of disturbance, tires and other larger containers were approached cautiously and the cup was immersed fast at the water surface instead of slowly “scooping” the water. For smaller containers the water was transferred to pans for immature stages collection. Water in tires and containers of which the opening was too narrow was sucked up with a pipette. Late instars' (3rd and 4th) larvae were killed in hot water and transferred to 70% ethanol for at least 2 hours and then to 95% ethanol for at least 2 hours. The larvae were then transferred to watch glass containing xylene and mounted on microscopic slides using Canada Balsam on microscopic slides. All samples were transported to the Biology Department, Dire Dawa University's laboratory, for identification. All late instars and adults which emerged from pupae were carefully identified to species under a microscope, using identification keys [22, 23]. The number and species of mosquito larvae and pupae from each tire and container were recorded and compared.

In order to carry out the survey, consent was obtained from the health authorities and households of Dire Dawa city.

### 2.3. Data Analysis

The larval survey data were calculated and analyzed in terms of different larval survey techniques like house index (HI), container index (CI), and breteau index (BI). The calculation of larval indices is based on the following mathematical formulae:

(1) House  Index  HI=Number  of  houses  infestedTotal  number  of  houses  inspected×100,Container  Index  CI=Number  of  positive  containers  infestedTotal  number  of  containers  inspected×100,Breteau  Index  BI=Number  of  positive  containersTotal  number  of  houses  inspected×100.

## 3. Results

A total of 301 houses were surveyed for the presence of artificial breeding containers for* Aedes* mosquitoes, and, of these, 208 houses were found to contain positive containers. Overall 750 artificial containers were inspected among which 405 containers were found positive for mosquito larvae. These were 135 (33.33%) tires, 65 (16.04%) barrels, 98 (24.19%) plastic drums, 77 (19.01%) jerricans, 5 (1.23%) mud pots, 2 (0.49%) flower pots, 5 (1.23%) discarded sinks, 11 (2.71%) buckets, 2 (0.49%) plastic bowls, 2 (0.49%) dustbins, 2 (0.49%) polythene sheets, and 1 (0.24%) discarded excavator (Table 1). Most of the artificial water storage containers were located outdoors (93.06%), were uncovered or partially covered (87.6%), and were totally or partially sun exposed (67.86%). The residents mostly store clean rain water (72.66%) for washing clothes as compared to tap water.

Larvae and pupae were found in all the identified containers; however, mosquitoes preferred to breed in tires (33.33%), barrels (16.04%), plastic drums (24.19%), and jerricans (19.01%) as compared to the others. Barrels, plastic drums, and jerricans are used mostly for storage of water for domestic use. Mosquitoes greatly breed in water holding tires at tire repair sites, discarded tires in different areas, and tires used for cloth washing in Peri-homesteads. Mud pots, discarded sinks, polythene sheet, discarded vehicle parts, plastic bowl, and buckets may store rain water in some localities and were serving for breeding. In some houses, dustbins hanged at the gate stored rain water and served for mosquito breeding.

As shown in Table 2, 1873 immatures were collected and identified from 405 containers. Among these, 1580 were* Ae. aegypti* and the remaining were* Culex* mosquitoes.* Aedes aegypti* bred in all types of water holding container even if it prefers some of the containers than the others. Most of the mosquito larvae were collected from containers containing rain water. Those containers which stored only tap water did not contain mosquito larvae, though they harbored mosquito larvae when they were mixed with rain water.

The results of the commonly used larval indices (house, container, and breteau index) are depicted in Table 3. HI, CI, and BI ranged between 33.33 and 86.15, between 23.18 and 73.91, and between 56.52 and 188.88, respectively, at different locations in the town. These indices showed that there was high infestation of artificial water containers by mosquito larvae which may cause an outbreak of dengue.

## 4. Discussion

The common breeding habitats observed in the study area were tires, barrels, plastic drums, and jerricans. The majorities of the residents in Dire Dawa store tap and rain water in containers for domestic use. Storing tap and rain water is common practice due to irregular supply and preference of rain water for laundry purpose. A study in Tirunelveli district, India, showed that, due to poor rainfall and shortage of water supply, the residents stored water in various containers for long duration and these containers constituted the major mosquito breeding sources [24]. Containers that retained water for long periods of time make good or suitable breeding habitats for mosquitoes such as the artificial containers [25, 26]. In Dar es Salaam, water storage often occurs in the presence of piped water systems because of intermittent water supply and due to the necessity of collecting supplementary rainwater [27]. In Dire Dawa,* Ae. aegypti* and* Culex* were found breeding in different water holding artificial containers.* Culex* species are mostly found in association with* Ae. aegypti* in tires and containers that contain leaf litter which are located under the shade especially tree shades. However, there were more* Ae. aegypti* mosquitoes breeding in area with high vegetation cover [27]. The coexistence of* Ae. aegypti* and* Culex* mosquitoes in households is likely attributable to the abundance of suitable containers that are favorable to all container-breeding mosquitoes and the availability of shade and sufficient organic material for larval feeding [28]. On the other hand,* Cx. quinquefasciatus* was found breeding only in plastic containers with polluted water [29]. Among mosquitoes that breed (exclusively or not) in artificial containers* Ae. aegypti*,* Ae. albopictus*, and* Culex pipiens* complex are highly abundant [15]. In spite of this, in our study,* Ae. aegypti* was found to be the most dominant species breeding in artificial containers. The containers were abundantly located close to human habitation and were potentially more durable than natural containers [30]. In our study, no* Anopheles* mosquito larvae were collected. However, in Nicholas County, West Virginia, anopheline mosquitoes also inhabited waste tires [31].

The types of the containers, water quality, and conditions of water containers are important for breeding [29]. All the identified* Aedes* were* Ae*.* aegypti* in our study area. Studies in urban forest in Rio de Janeiro [32], Central Africa [33], and Laos [34] showed* Ae. aegypti* was strongly associated with urban environments.

Water chemistry of aquatic habitats may also play a critical role in determining the survival rate of mosquitoes [29, 35].* Aedes aegypti* exhibits a great deal of specialization in breeding site selection and consequently the distribution of this species is limited by those sites [36]. Since the presence of water in containers is probably the most important factor in determining the breeding of mosquitoes, especially* Aedes* and* Culex* species, a mosquito control programme should be established in Dire Dawa. For the control of container breeding mosquitoes it is possible to use different methods in integration and these include covering water holding containers [27, 34], using appropriate biological control agents [27], public health education [24, 25, 37], creating knowledge and awareness of the residents on mosquito-borne diseases [37], eliminating water-filled unused containers [24, 25], draining of containers once a week [34], and proper waste management system for all housing areas [25]. However, targeting specific types of water-holding containers would enable a more focused approach to vector control than attempting to eliminate all water-holding containers [38].

## 5. Conclusion

In the study area, the community store water in different containers for long period of time for the domestic use. In addition to domestic containers, different discarded containers and tires hold rain water for long period time. This enables* Ae. aegypti* to breed in these containers. As our study showed, most of the containers were infested with this mosquito species which may serve as vector of dengue disease. From this investigation, it is clear that there are many chances of mild dengue viral infection spreading in the sampling location. However, to determine whether this mosquito is transmitting disease or not by looking for the virus in the mosquitoes needs further investigation.

This study involved only collection and identification of mosquito larvae from tires, household containers, and discarded water holding materials so that it needs further investigation to look for mosquito larvae in natural water holding containers and larger water tanks. There has to be a viral isolation through collecting the adult females to look if they harbor the dengue disease pathogen. It also needs awareness creation of the population not to be affected by the disease in case epidemic may occur. Since this study was only in Dire Dawa town, it should also be in the surrounding kebeles to identify the foci of the disease. In containers containing tap water, mosquitoes larvae were not abundant and were found in tap water mixed with rain water. This indicated the need to study water chemistry to know the reason behind the fact that mosquitoes were not reproducing in containers with tap water only.

## Figures and Tables

**Table 1:** Mosquito larval breeding containers and their characteristics in Dire Dawa.

Type of container	Number of containers inspected	Number of positive containers	Location		Sun exposure			Lid status			Water type			Water status	
		*N*	Outside	Inside	Low	Partial	High	Unlidded	Partial	Lidded	Rain	Tap	Mix	Clean	Polluted
Tire	276	135	276	—	78	56	142	231	35	10	259	15	2	263	13
Barrel	78	65	73	5	29	34	15	33	39	6	57	14	7	56	22
Plastic drum	116	98	103	13	35	68	13	31	79	6	87	11	18	109	7
Jerrican	143	77	121	22	39	83	21	37	44	62	52	69	22	129	14
Mud pot	8	5	6	2	5	2	1	1	4	3	6	—	2	7	1
Flower pot	28	2	28	—	12	9	7	28	—	—	12	—	16	22	6
Discarded sink	33	5	33	—	9	24	—	33	—	—	33	—	—	27	6
Buckets	35	11	29	6	13	16	6	29	—	6	15	4	16	31	4
Plastic bowl	19	2	15	4	10	9	—	19	—	—	10	—	9	14	5
Dustbin	8	2	8	—	6	1	1	8	—	—	8	—	—	3	5
Polythene sheet	5	2	5	—	4	1	—	5	—	—	5	—	—	2	3
Discarded excavator	1	1	1	—	1	—	—	1	—	—	1	—	—	—	1

Total	750	405	698	52	241	303	206	456	201	93	545	113	92	663	87

**Table 2:** Mosquitoes identified from larvae collected from artificial containers in Dire Dawa.

Type of container	Number of containers inspected	Number of positive containers (%)	Species of mosquitoes	
			*Ae. aegypti* (%)	*Culex* (%)
Tire	276	135 (33.33)	221 (13.98)	146 (49.82)
Barrel	78	65 (16.04)	371 (23.48)	56 (19.11)
Plastic drum	116	98 (24.19)	659 (41.70)	28 (9.55)
Jerricans	143	77 (19.01)	144 (9.11)	12 (4.09)
Mud pot	8	5 (1.23)	54 (3.41)	0
Flower pot	28	2 (0.49)	2 (0.12)	5 (1.70)
Discarded sink	33	5 (1.23)	5 (0.31)	16 (5.46)
Buckets	35	11 (2.71)	12 (0.75)	0
Plastic bowl	19	2 (0.49)	29 (1.83)	0
Dustbin	8	2 (0.49)	35 (2.21)	0
Polythene sheet	5	2 (0.49)	46 (2.91)	0
Discarded excavator	1	1 (0.24)	2 (0.12)	30 (10.23)

Total	750	405	1580	293

**Table 3:** Larval indices and distribution of *Aedes aegypti* breeding habitats at different locations in Dire Dawa.

Location (site)	Total houses	Positive houses	Total containers	Positive containers	HI	Cl	BI
Dipo	59	45	146	94	76.27	64.38	159.32
Sabean	65	56	138	102	86.15	73.91	156.92
Number One	36	19	117	68	52.77	58.11	188.88
Gende Kore	38	26	89	56	68.42	62.92	147.36
Addis Ketema	36	25	126	38	69.44	30.15	105.55
Dechatu	23	12	38	13	52.17	34.21	56.52
Afetesa	16	9	31	12	56.25	38.70	75.00
Lege Hare	19	13	69	16	68.42	23.18	84.21
Gende Gerada	9	3	16	6	33.33	37.50	66.66

Total	301	208	750	405	69.10	54.00	134.55

HI, house index; Cl, container index; BI, breteau index.
